# Intratumoral FoxP3^+^Helios^+^ Regulatory T Cells Upregulating Immunosuppressive Molecules Are Expanded in Human Colorectal Cancer

**DOI:** 10.3389/fimmu.2017.00619

**Published:** 2017-05-26

**Authors:** Azharuddin Sajid Syed Khaja, Salman M. Toor, Haytham El Salhat, Bassam R. Ali, Eyad Elkord

**Affiliations:** ^1^Cancer Research Center, College of Science and Engineering, Qatar Biomedical Research Institute, Hamad Bin Khalifa University, Qatar Foundation, Doha, Qatar; ^2^Department of Medical Microbiology and Immunology, College of Medicine and Health Sciences, United Arab Emirates University, Al Ain, United Arab Emirates; ^3^Oncology Department, Al Noor Hospital, Abu Dhabi, United Arab Emirates; ^4^Oncology Department, Tawam Hospital, Al Ain, United Arab Emirates; ^5^Department of Pathology, College of Medicine and Health Sciences, United Arab Emirates University, Al Ain, United Arab Emirates; ^6^Institute of Cancer Sciences, University of Manchester, Manchester, United Kingdom

**Keywords:** colorectal cancer, regulatory T cells, Forkhead box protein 3, Helios, immune checkpoint receptors, tumor microenvironment

## Abstract

Regulatory T cells (Tregs) can be antitumorigenic or pro-tumorigenic in colorectal cancer (CRC) depending on the presence of different Treg subsets with various immunosuppressive molecules. Some studies reported the phenotypic characteristics of tumor-infiltrating immune cells in CRC, but limited studies have focused on the co-expression of suppressive molecules on immune cells. The aim of this study was to characterize immune cells in the tumor microenvironment (TME), compared to paired adjacent non-tumor colon tissue of CRC patients. Additionally, we investigated co-expression of immunosuppressive molecules on different Treg subsets in the TME, normal colon tissue, and peripheral blood of CRC patients and healthy donors. In this preliminary study, we report that the majority of CD3^+^ T cells in the TME are CD4^+^ T cells with high co-expression of programmed death 1 (PD-1)/cytotoxic T-lymphocyte-associated protein 4 (CTLA-4) and PD-1/CD39 molecules. Levels of CD4^+^FoxP3^+^Helios^+^ Tregs were significantly increased in the TME. Furthermore, we observed increased levels of PD-1/CTLA-4 and PD-1/CD39 co-expressing cells within FoxP3^+^Helios^+^ and FoxP3^+^Helios^−^ Treg subsets, indicative of their potent immunosuppressive potential. These results suggest synergistic associations between PD-1/CTLA-4 and PD-1/CD39 in dampening T-cell activation and function along with suppressing tumor-specific immune responses, suggesting that dual blockade of these molecules could be a more effective strategy for inducing antitumor immune responses in CRC.

## Introduction

Regulatory T cells (Tregs) play important roles in maintaining immune homeostasis and preventing autoimmunity in normal physiological conditions by suppressing self-reactive T cells. However, under pathophysiological conditions such as cancer, their suppressive functions promote the growth and progress of disease by hindering antitumor immune responses (1). Earlier identified as CD4^+^CD25^+^ T lymphocytes (2), the majority of Tregs were later characterized by expression of Forkhead box protein 3 (FoxP3), a master regulatory transcriptional factor (3), which is critical for the development and suppressive functions of Tregs (4). Tregs are heterogeneous in their development and can be grouped into two major subsets; natural or thymic-derived Tregs (tTregs) and peripheral-induced Tregs (pTregs). However, specific markers, which could reliably distinguish between the two subsets, are elusive. Helios, a member of the Ikaros transcription factor family, was proposed as a marker to distinguish tTregs from pTregs (5), but this work was later questioned by several other studies (6–8). Helios, however, is shown to be a marker for T cell activation and proliferation (9), and its expression is essential for the stable Treg inhibitory activity (10). We have recently shown that Helios is a marker of activated Tregs expressing immunosuppressive molecules GARP/latency-associated peptide (LAP) (11) and that co-expression of Helios and FoxP3 with GARP/LAP can be used to identify expanded Treg subset in cancer patients (12).

The immunosuppressive mechanisms of Tregs are not fully understood but immune checkpoint receptors [ICR; such as programmed death 1 (PD-1) and cytotoxic T-lymphocyte-associated protein 4 (CTLA-4)], immunosuppressive molecules such as CD39 and LAP have been shown to play critical roles in their functions (13, 14). CTLA-4 (CD152) is constitutively expressed by Tregs where it enhances Treg suppressive functions. Studies in human and murine models showed that blocking CTLA-4 signaling by monoclonal antibodies increased proliferation of T effector cells and impaired Treg suppressive activity (15, 16). PD-1 (CD279), like CTLA-4, is highly expressed on Tregs and helps in Treg immunosuppression (17), and blocking PD-1 pathway with anti-PD-1 monoclonal antibody prevented Treg-mediated suppression of effector T cells *in vitro* (18). Several other studies have also reported the elevated levels of CD39, an ectonucleotidase, on the cell surface of Tregs (13, 19, 20). CD39 is involved in the conversion of ATP to adenosine which is a potent immunosuppressive molecule and blocks proliferation and function of effector T cells (21). Schuler et al. have characterized Treg subsets based on the presence or absence of CD39, FoxP3, and CD25, and showed that CD4^+^CD39^+^FoxP3^+^CD25^+^ Tregs suppressed proliferation of effector T cells (22).

Regulatory T cells have been implicated in progression of cancers due to their ability to suppress other T lymphocytes and therefore are considered to be a major barrier for antitumor immunotherapies. Several studies have shown Treg infiltration at tumor sites in various cancers including colorectal cancer (CRC) and their enrichment within tumor-infiltrating lymphocytes (TILs) (1, 20, 23, 24). CRC is one of the most common malignancies worldwide (25). CRC is mainly an inflammation-associated cancer, and Tregs are expanded in tumor microenvironment (TME) and play important roles in the pathogenesis of CRC (26). Though accumulation of FoxP3^+^ Tregs in majority of human carcinomas is associated with tumor growth and poor outcome, some studies in CRC have shown the presence of Tregs in tumors is associated with better outcome (27), suggesting that in CRC Treg functions might be antitumorigenic by suppressing inflammation at the early stages of the disease. However, some other studies have shown that Tregs in colorectal tumor tissue (TT) might contribute to disease progression by suppressing antitumor immune responses (28, 29). These seemingly contradicting results suggest the presence of different Treg subpopulations with varying degrees of suppression (30). Saito et al. (31) showed that these functionally heterogeneous Treg subpopulations affected prognosis of CRC in different ways, as CD45RA^−^FoxP3^lo^ non-Treg subset secreted pro-inflammatory cytokines and patients with significant accumulation of this Treg subset had better prognosis compared to those with highly suppressive CD45RA^−^FoxP3^hi^ Treg subset. They also reported that pro-inflammatory FoxP3^lo^ Tregs have lower expression of CTLA-4 and TIGIT (T cell immunoreceptor with Ig and ITIM domains), indicating that immunosuppressive molecules on T cells and Tregs affect the outcome of CRC disease. Therefore, studies focusing on characterization of Tregs in the colorectal TME with potential implications for CRC treatment are warranted.

In this preliminary study, we investigated the levels and phenotypes of immune cells infiltrating colorectal TT and compared them with adjacent non-tumor normal tissue (NT) and peripheral blood from the same patients. We also characterized different subsets of TILs and assessed the expression of PD-1, CTLA-4, and CD39 on different FoxP3 and Helios Treg subsets. We found that CD4^+^FoxP3^+^Helios^+^ Tregs are expanded in the TME of CRC patients compared with normal tissue and peripheral blood. Additionally, we observed increased frequency of PD-1/CTLA-4 and PD-1/CD39 co-expressing cells within FoxP3^+^Helios^+^ and FoxP3^+^Helios^−^ Treg subsets. CD4^+^FoxP3^+^Helios^+^ and CD4^+^FoxP3^+^Helios^−^ T cells expressed similar relative percentages of ICRs/CD39, but the absolute percentages were significantly higher in CD4^+^FoxP3^+^Helios^+^ Treg subset, indicating the potent immunosuppressive potential of CD4^+^FoxP3^+^Helios^+^ Treg subset.

## Materials and Methods

### Human Tissue and Blood Samples and Peripheral Blood Mononuclear Cell (PBMC) Isolation

Tumor and paired, adjacent non-tumor colon tissues were obtained from 12 CRC patients who underwent surgical excision of primary tumors at Tawam Hospital, Al Ain and Al Noor Hospital, Abu Dhabi, UAE. None of the patients included in this study received any treatment prior to surgery. Tumor samples were assessed for Dukes staging and TNM staging by senior pathologists. Additionally, around 20 mL of peripheral blood was collected in tubes with heparin from cancer patients (*n* = 12) before surgery. As controls peripheral blood samples from healthy donors (HDs; *n* = 9) were used. Table 1 shows the clinical and pathological characteristics of all participating individuals. Ethical approval (Protocol no. 13/23-CRD 244/13) was obtained from Al Ain Medical District Human Research Ethics Committee. All participating individuals signed written informed consent prior to collection of samples. All experiments were performed in accordance with relevant guidelines and regulations.

**Table 1:** **Characteristic features of study populations**.

	HD	CRC
Number	9	12 (12)^b^
Age (median)	27 (19–45)^a^	44 (36–74)^a^
Gender (male:female)	7:2	5:7
**TNM stage**		
I		1 (1)^b^
II		5 (5)^b^
III		5 (5)^b^
IV		1 (1)^b^
Tumor size (in cm)		4.3 (0.2–9.5)^a^
Lymph node invasion		6
**Histological grade**		
Well/moderate		11
Poor/undifferentiated		1

*HD, healthy donor; CRC, colorectal cancer*.

*^a^Data shown represent median (range)*.

*^b^Samples taken from patients for investigating tissue-infiltrating immune cells*.

Peripheral blood mononuclear cells were isolated from whole blood by density gradient centrifugation using Histopaque-1077 (Sigma-Aldrich, Dorset, UK). After isolation, PBMCs were frozen in cryovials at a density of five million cells per 1 ml freezing media (50% FBS, 40% RPMI 1640 media, and 10% DMSO). For all flow cytometry staining, cryopreserved PBMCs were used.

### Cell Isolation from Colorectal Tumor and Non-Tumor Normal Tissues by Enzyme Disaggregation

Cell suspensions were obtained from TTs and paired non-tumor normal tissues (NT) by enzymatic digestion, as previously described (32). Briefly, surgically removed fresh colon TT and NT were cut into small pieces and suspended in RPMI media with 1% penicillin/streptomycin and enzyme cocktail (1 mg/ml collagenase, 100 µg/ml hyaluronidase type V and 30 IU/ml of deoxyribonuclease I, all from Sigma-Aldrich, UK) and incubated at 37°C under slow rotation for 60 min. The resulting cell suspension was then filtered through a 100-µm BD Falcon cell strainer (BD Biosciences, Oxford, UK), washed twice, and resuspended in complete media. Immune cells, isolated from TT (referred as TILs) and NT [referred as non-tumor-infiltrating lymphocytes (NILs)], were characterized by appropriate gating by flow cytometric analysis.

### Multicolor Flow Cytometry

#### Surface Staining for Identification of Immune Cells

Tumor-infiltrating lymphocytes and NILs were washed and suspended in 100-µL staining solution (PBS with 2% serum and 0.1% sodium azide). Cells were then incubated with FcR blocker (Miltenyi Biotec, Bergisch Gladbach, Germany) for blocking Fc receptor and 7AAD dye was used to discriminate between live and dead cells. For identification of immune cells, TILs and NILs were stained with cell surface antibodies CD45-FITC (clone HI30, eBioscience, San Diego, CA, USA), CD3-APC.H7 (clone SK7, BD Biosciences, Oxford, UK), CD4-Alexa Fluor 700 (clone RPA-T4, BioLegend, San Diego, CA, USA), CD25-PE/Cy7 (clone M-A251, BioLegend), PD-1-PE/Dazzle™ 594 (clone EH12.2H7, BioLegend), and CD39-PerCP/Cy5.5 (clone A1, BioLegend) for 30 min at 4°C. After staining, cells were washed and cell pellet was resuspended in flow cytometry staining buffer (eBioscience).

#### Surface and Intracellular Staining

Following blocking with FcR blocker, PBMCs, TILs, and NILs were stained with Fixable Viability Dye eFluor^®^ 660 (FVD 660; eBioscience) according to the manufacturer’s protocol to gate for live cells. Cells were then surface labeled with antibodies CD3-APC-H7 (BD Biosciences), CD4-Alexa Fluor 700 (BioLegend), LAP-PE (clone TW4-2F8; BioLegend), and PD-1- PE/Dazzle™ 594 (BioLegend) for 30 min at 4°C. After washing with staining solution, cells were fixed and permeabilized using fixation/permeabilization buffer (eBioscience) at 4°C for 45 min after thorough vortexing. Cells were then washed twice with permeabilization wash buffer (eBioscience) and blocked using mouse serum (Sigma-Aldrich) and rat serum (eBioscience) for 10 min. Cells were then stained with antibodies CTLA-4-PerCP-eFluor^®^ 710 (clone 14D3; eBioscience), FoxP3-PE-Cyanine7 (clone PCH101, eBioscience), and Helios-FITC (clone 22F6, BioLegend) for 30 min at 4°C. Cells were washed twice with permeabilization wash buffer (eBioscience) and were resuspended in flow cytometry staining buffer for flow cytometric analyses. Data were acquired with a BD FACSCanto II flow cytometer using BD FACSDiva software (BD Bioscience) and analyzed on BD FACSuite software (BD Biosciences). Out of 12 CRC samples, we did not consider intracellular staining data from 2 samples since no cell viability dye was used for these.

### Statistical Analyses

All the statistical analyses were performed using GraphPad Prism 5.0 software (GraphPad Software, USA) or Microsoft Excel (Microsoft Corporation, Washington, DC, USA). Shapiro–Wilk normality test was used to find out if the data were normally distributed. Paired/Wilcoxon matched-pairs signed rank test or unpaired/Mann–Whitney tests were used to study the differences within groups or between groups, respectively. Welch’s *t*-test was applied for unequal sample sizes between the groups. Correlations between different markers were calculated using non-parametric Spearman’s correlation test. For all the analyses, *p* value <0.05 was considered statistically significant.

## Results

### Characterization of Tumor-Infiltrating T Cells in CRC

Presence of TILs was examined in fresh TTs from 12 CRC patients and their levels were compared with NILs from adjacent normal tissue from the same patients. Live cells were gated first using 7AAD viability dye. Tumor-infiltrating leukocytes were identified using CD45 antibody, and staining with CD3, CD4, and CD8 antibodies was used to identify T lymphocyte subsets (Figure S1A in Supplementary Material). We detected a significant infiltration of CD45^+^CD3^+^ T lymphocytes in TT compared with NT (TT—10.5 ± 2.8% vs. NT—1.9 ± 1.2%; Figure S1B in Supplementary Material). When characterized further, we found that within CD3^+^ cells, the majority of T cells infiltrating tumors were CD3^+^CD4^+^ T cells (TT—56.6 ± 2.9% vs. NT—39.6 ± 6.7%; Figure S1B in Supplementary Material), whereas the relative percentages of infiltrating CD3^+^CD8^+^ T cells were similar when compared between TT and NT (TT—37.2 ± 3.2% vs. NT—32.0 ± 6.3%; Figure S1B in Supplementary Material). Moreover, there was a marked increase in frequency of CD45^+^CD3^−^ cells within TT compared with NT (Figure S1A in Supplementary Material). These cells could be of myeloid origin and might play important roles in CRC pathogenesis.

**Figure 1:**
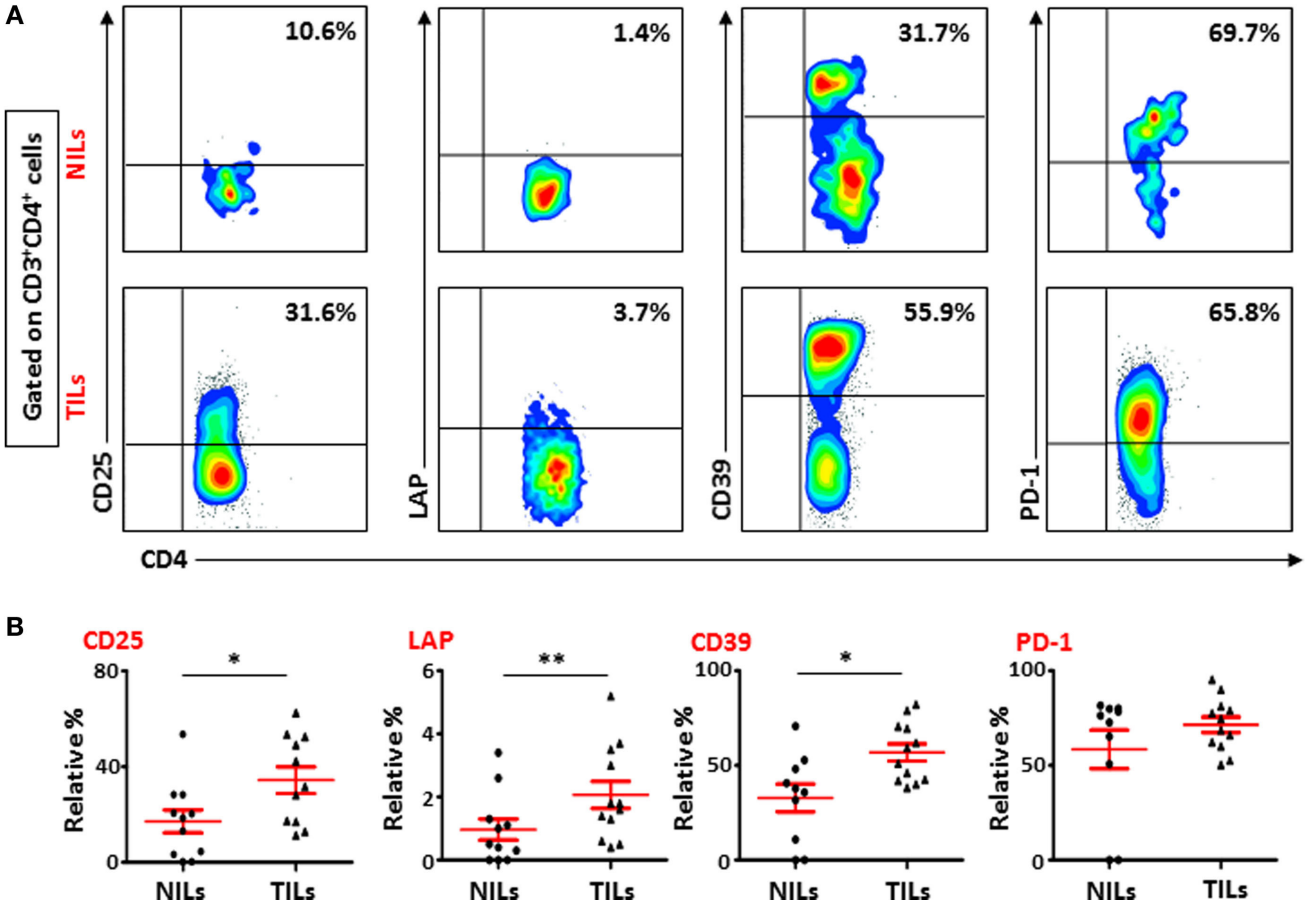
**Phenotypic characterization of CD4^+^ T cells in non-tumor-infiltrating lymphocytes (NILs) and tumor-infiltrating lymphocytes (TILs)**. Freshly isolated NILs and TILs were stained for CD25, LAP, CD39, and PD-1 surface markers and their frequencies were calculated in CD4^+^ T cells. **(A)** Representative flow cytometric plots for these markers in NILs and TILs from a cancer patient. **(B)** Scatter plots showing the differences between NILs and TILs.

To further study the phenotypic characteristics of these intratumoral T cells, we evaluated the expression of CD25, immunosuppressive molecules such as LAP and CD39, and PD-1 on surface of CD4^+^ T lymphocytes within TILs and NILs. Representative flow cytometric plots showing expression of CD25, LAP, CD39, and PD-1 on CD4^+^ T lymphocytes in NILs and TILs are shown in Figure 1A. CD25 was highly expressed in CD4^+^ T cells in TILs compared with NILs (TT—34.4 ± 5.6% vs. NT—17.1 ± 4.8%; Figure 1B). The percentages of CD4^+^ T cells expressing immunosuppressive molecules such as CD39 (TT—56.8 ± 5.6% vs. NT—32.8 ± 7.3%) and LAP (TT—2.1 ± 0.4% vs. NT—1.0 ± 0.3%; in non-activated state) were also significantly elevated in TILs compared with NILs (Figure 1B). There was no difference in the levels of PD-1 expressing CD4^+^ T cells between TILs and NILs (TT—71.5 ± 4.1% vs. NT—58.5 ± 10.2%; Figure 1B). However, levels of PD-1 expressing CD8^+^ T cells were increased significantly in TT compared with NT (TT—61.5 ± 5.3% vs. NT—33.1 ± 7.2%; Figures S2A,B in Supplementary Material). Within CD8^+^ population, there were no differences in the levels of cells expressing CD25 (TT—13.5 ± 3.0% vs. NT—9.1 ± 3.0%), LAP (TT—0.8 ± 0.2% vs. NT—0.3 ± 0.2%) and CD39 (TT—47.6 ± 6.8% vs. NT—40.3 ± 8.2%) between TILs and NILs (Figures S2A,B in Supplementary Material). Within TILs, CD4^+^ T cells expressed significantly higher levels of CD25 and LAP compared with CD8^+^ T cells, whereas there were no differences in the expression levels of CD39 and PD-1 expressing cells between these populations (Figure S2C in Supplementary Material).

### CD4^+^ T Cells Co-Expressing PD-1/CTLA-4 and PD-1/CD39 Are Elevated in TILs

**Figure 2:**
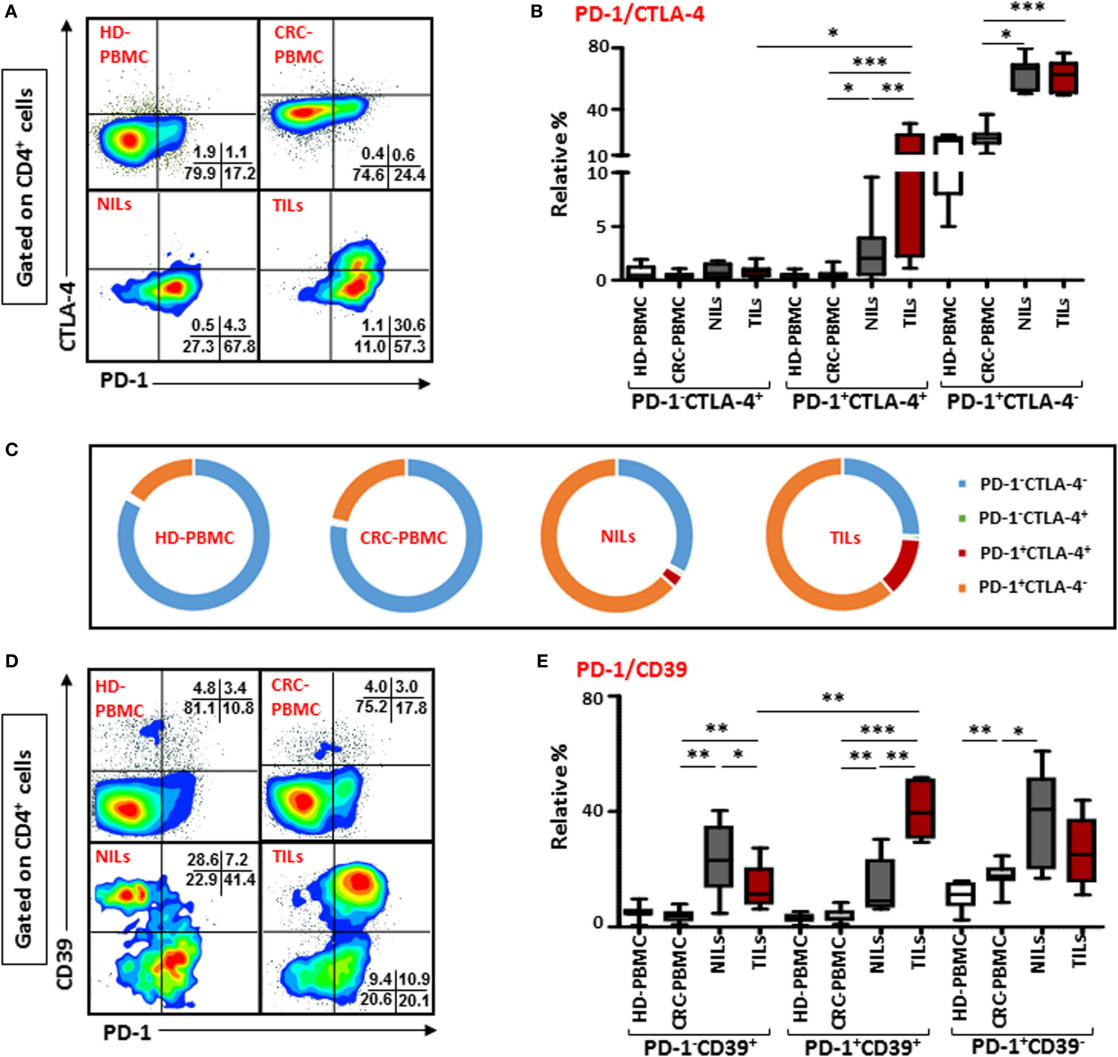
**Co-expression of PD-1/CTLA-4 and PD-1/CD39 in CD4^+^ T cells in healthy donor (HD) peripheral blood mononuclear cells (PBMCs), colorectal cancer (CRC) patients PBMCs, non-tumor-infiltrating lymphocytes (NILs), and tumor-infiltrating lymphocytes (TILs)**. PBMCs from HD and CRC patients, NILs, and TILs were stained for CD3, CD4, PD-1, and CD39. After fixation and permeabilization, cells were stained for CTLA-4 expression. Live cells were gated using Fixable Viability Dye 660. Representative flow cytometric plots showing PD-1 and CTLA-4 in CD4^+^ T cells **(A)** and whisker plots **(B)** showing differences in their expression in HD-PBMC, CRC-PBMC, NILs, and TILs. **(C)** Pie charts show the relative percentages of PD-1 and CTLA-4 in CD4^+^ T cells. Representative flow cytometric plots showing different subpopulations of PD-1 and CD39 in CD4^+^ T cells **(D)** and whisker plots **(E)** showing differences in their expression in different samples.

There was no difference in the levels of PD-1 expressing CD4^+^ T cells between TILs and NILs, but when we examined its co-expression with intracellular CTLA-4, another ICR, there was a significant increase in the levels of cells co-expressing PD-1 and CTLA-4 infiltrating TT (12.7 ± 3.7%), compared with their levels in NT (2.8 ± 1.1%) and circulation (0.5 ± 0.1%) in CRC patients (Figures 2A,B). Normal tissue also has significantly higher frequencies of CD4^+^ T cells co-expressing PD-1^+^CTLA-4^+^ compared with their levels in peripheral blood, indicating that NT-resident CD4^+^ T cells have increased levels of ICRs. There was no difference between the levels of PD-1 and CTLA-4 co-expressing CD4^+^ T cells in circulation when compared between HD (PBMC—0.4 ± 0.1%) and CRC patients. We also observed significant accumulation of CD4^+^ T cells expressing only PD-1 in TT (61.2 ± 3.4%) and NT (NT—63.5 ± 3.6%), compared with their levels in peripheral blood (PBMC—21.6 ± 1.9%). The relative percentages of CD4^+^PD-1^+^CTLA-4^+^ T cells in peripheral blood of HDs and CRC patients were negligible, where majority of cells were either PD-1^−^CTLA-4^−^ or expressed only PD-1, whereas in NILs and TILs, CD4^+^PD-1^+^CTLA-4^−^ T cells increased significantly (Figure 2C). TILs also had higher levels of CD4^+^ T cells expressing PD-1^+^CTLA-4^+^. Additionally, CD8^+^ T cells co-expressing PD-1 and CTLA-4 were increased in TILs (2.5 ± 0.6%), compared with NILs (0.5 ± 0.2%) and peripheral blood (0.1 ± 0.0%; Figure S3A in Supplementary Material). Clearly, in TILs, CD4^+^ T cells had significantly higher expression of PD-1^+^CTLA-4^+^ than CD8^+^ T cells (Figure S3C in Supplementary Material).

Co-expression of PD-1 with CD39 on CD4^+^ T cells were also compared between TILs, NILs, and PBMC from patients and PBMC from HD. There was no difference in their expression levels in PBMC from HDs (2.9 ± 0.5%) and CRC patients (3.7 ± 0.8%), but there was a significant infiltration of CD4^+^ T cells co-expressing PD-1 and CD39 in TT (40.5 ± 4.5%) compared with NT (13.6 ± 4.5%) and peripheral blood (Figures 2D,E). Notably, levels of CD4^+^ T cells expressing only CD39 but not PD-1 were significantly higher in NT (23.9 ± 5.8%) compared with TT (13.4 ± 3.7%) and peripheral blood (3.7 ± 0.7%; Figure 2E). Within TILs, levels of CD4^+^ T cell co-expressing PD-1 and CD39 were relatively higher than cells expressing CD39 alone. The frequency of CD8^+^ T cells co-expressing PD-1 and CD39 were also significantly elevated in TT compared with NT and peripheral blood (Figure S3B in Supplementary Material). There was no difference in the levels of PD-1^+^CD39^+^ between CD4^+^ and CD8^+^ T cells (Figure S3C in Supplementary Material).

### CD4^+^FoxP3^+^ Tregs Are Increased in CRC Tissue

**Figure 3:**
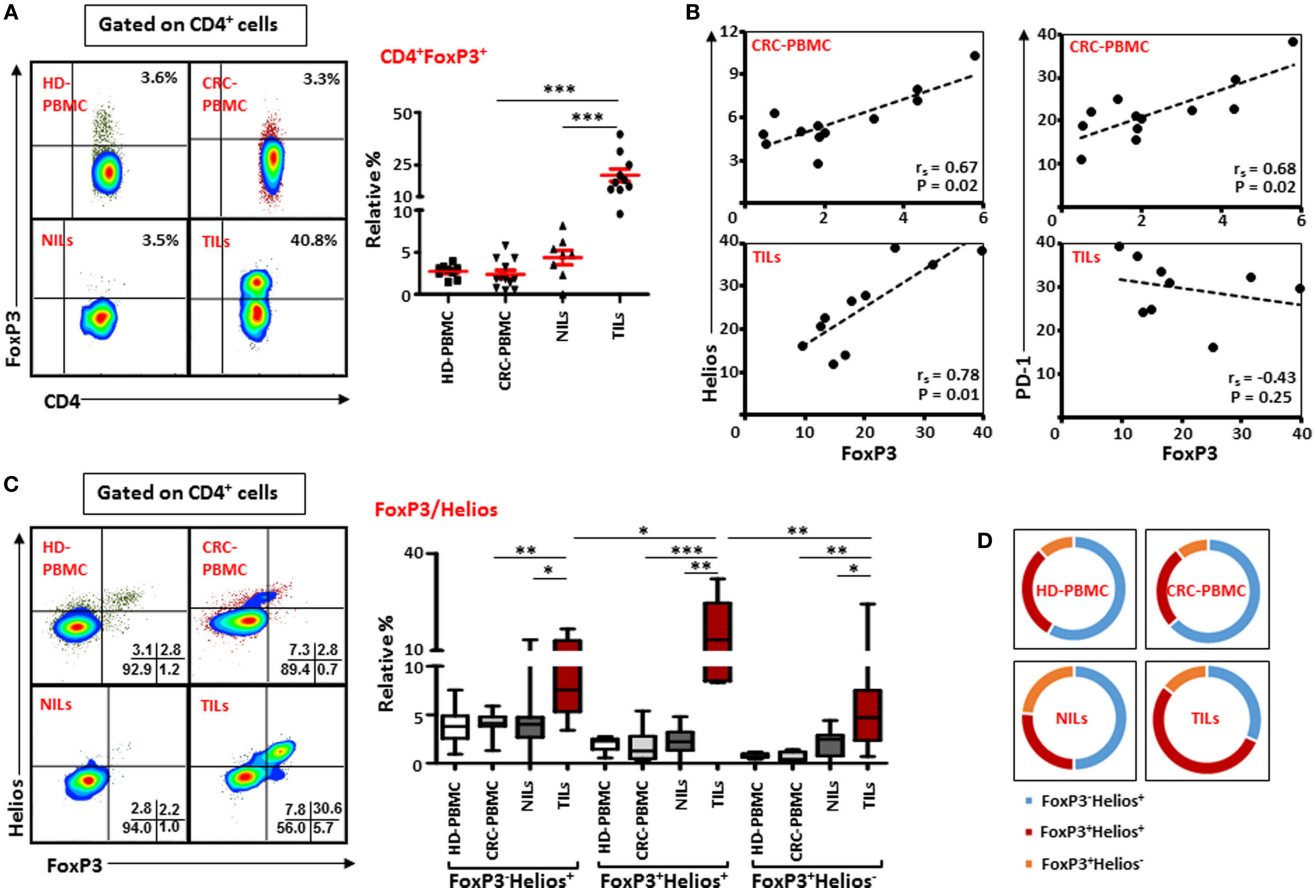
**FoxP3 and Helios expression in CD4^+^ T cells**. Peripheral blood mononuclear cells (PBMCs) from healthy donor and colorectal cancer (CRC) patients, non-tumor-infiltrating lymphocytes (NILs) and tumor-infiltrating lymphocytes (TILs) were stained for CD3 and CD4 followed by FoxP3 and Helios intracellular staining. Live cells were first gated using Fixable Viability Dye 660. Representative flow cytometric plots of FoxP3 staining are shown in **(A)**. Adjacent Scatter plot shows the differences between frequencies of regulatory T cells (Tregs) based on FoxP3 expression between different samples. **(B)** Non-parametric Spearman’s test was performed to investigate correlations between FoxP3, Helios, and PD-1 expressions in CRC-PBMCs and TILs. **(C)** Flow cytometric plots of FoxP3 and Helios co-expression in Tregs from different samples, and the adjacent whisker plot showing differences in various subsets of FoxP3 and Helios between them. **(D)** Pie charts show the relative percentages of three different FoxP3 and Helios Treg subsets.

Next, we investigated the frequency of Tregs within the heterogeneous population of TILs and NILs by examining the expression of FoxP3 on T cells. Similar to earlier reports (23, 26, 33), we found that within TT there was a marked increase in the level of CD4^+^FoxP3^+^ T cells compared with NT (TT—20.2 ± 2.9% vs. NT—4.4 ± 0.9%) and peripheral blood (2.4 ± 0.5%; Figure 3A). The proportion of Tregs increased slightly in NT compared to peripheral blood but the increase was not statistically significant (Figure 3A). In contrary to other studies on Treg levels in peripheral blood of CRC patients and HD (24, 29), we did not find any significant difference between relative percentages of Tregs expressing FoxP3 between CRC patients and HD (2.7 ± 0.3%; Figure 3A). We also investigated frequencies of FoxP3^+^ Tregs in different pathological stages and grades of CRC in the present patient cohort but did not find any differences in their levels between different stages or grades of the disease due to small sample size (data not shown).

### FoxP3 Is Positively Correlated with Helios Expression in Circulating and Intratumoral Tregs in CRC Patients

The suppressive functions of FoxP3^+^ Tregs can be regulated by the presence of different factors, such as Helios, CTLA-4, and PD-1 (10, 14, 34). Therefore, we investigated possible correlations between Helios, CTLA-4, and PD-1 expressions with FoxP3 expression on Tregs in peripheral blood, NILs, and TILs. We found FoxP3 expression was strongly and positively correlated with Helios expression within TILs [Spearman’s rank correlation coefficient (*r*_s_) = 0.782, *p* = 0.01; Figure 3B] and in circulation in cancer patients (*r*_s_ = 0.671, *p* = 0.02), suggesting that intratumoral and circulating Tregs have higher expression of FoxP3 with Helios. A similar positive correlation between FoxP3 and PD-1 was also observed in circulation in cancer patients (*r*_s_ = 0.678, *p* = 0.02). Interestingly, there was a negative association between levels of FoxP3 and PD-1 expression in TILs though it was not statistically significant (*r*_s_ = −0.43, *p* = 0.25). We did not find any correlation between FoxP3, CTLA-4, and CD39 in PBMCs, NILs, and TILs in the present patient cohort (data not shown).

### Intratumoral FoxP3^+^ Tregs Co-Express Helios

We then evaluated the co-expression of Helios and FoxP3 on Tregs from peripheral blood, NILs, and TILs of cancer patients. We observed that CD4^+^FoxP3^+^ Tregs in TILs expressed higher levels of Helios compared with their levels in NILs (TILs—16.6 ± 2.8% vs. NILs—2.2 ± 0.5%) and peripheral blood (1.7 ± 0.4%; Figure 3C). There was no significant difference in the levels of FoxP3^+^Helios^+^ Tregs between PBMCs of HD (2.0 ± 0.2%) and CRC patients. Within TT, frequencies of FoxP3^+^Helios^+^ Tregs were elevated compared with FoxP3^−^Helios^+^ and FoxP3^+^Helios^−^ Tregs (Figure 3C). Moreover, levels of FoxP3^−^Helios^+^ and FoxP3^+^Helios^−^ Treg subsets were significantly higher in TILs compared with NILs or PBMCs (Figure 3C). Among various FoxP3 and Helios Treg subsets, the proportion of FoxP3^−^Helios^+^ Treg subset was relatively high in PBMCs of HD and CRC patients, whereas FoxP3^+^Helios^+^ Treg subset proportion was highest in TILs (54%) compared to NILs (26.3%), PBMCs (26.2%) of cancer patients (Figure 3D).

### Intratumoral FoxP3^+^Helios^+^ and FoxP3^+^Helios^−^ Tregs Co-Express High Levels of PD-1/CTLA-4 and PD-1/CD39

**Figure 4:**
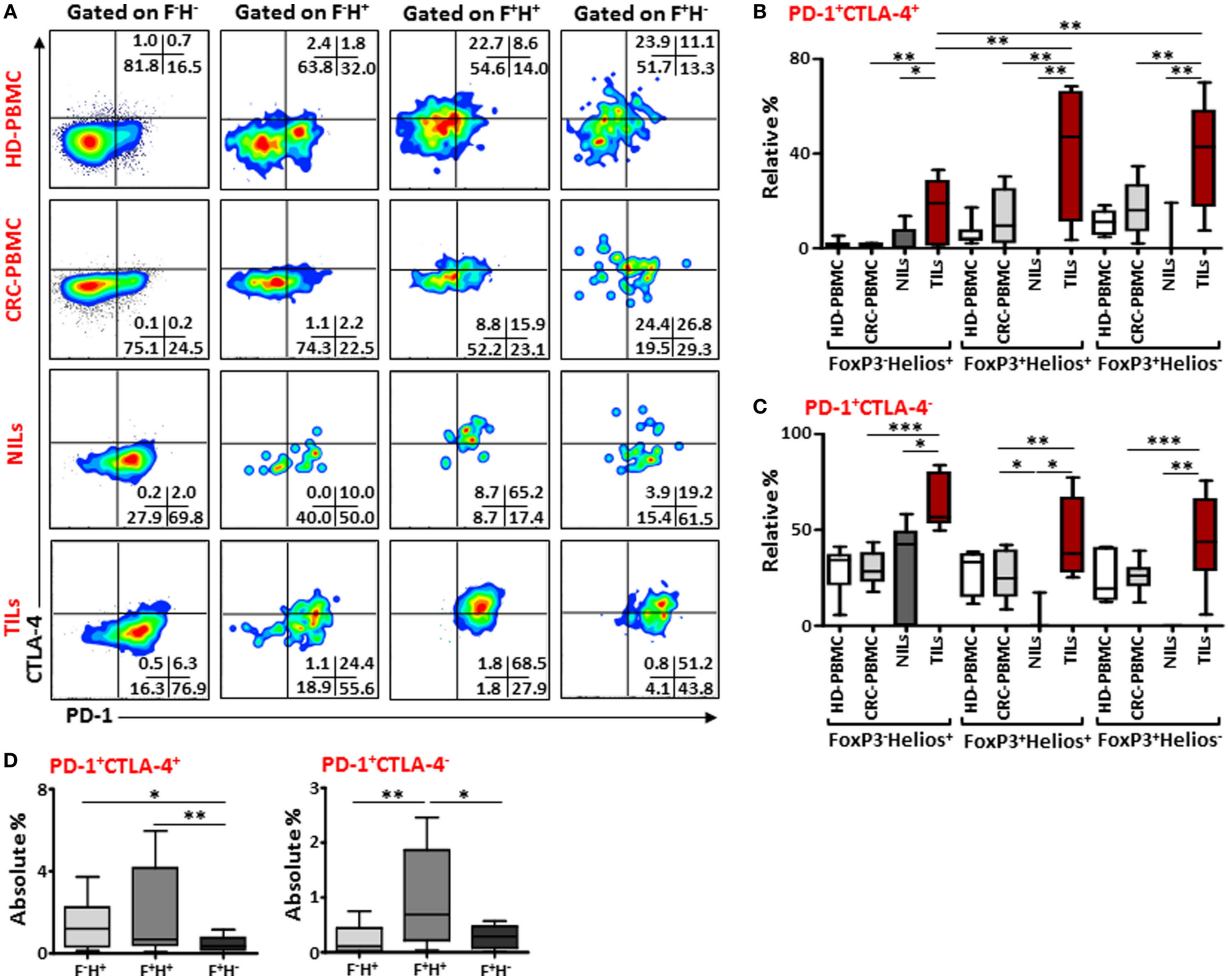
**PD-1 and CTLA-4 co-expression in different FoxP3 and Helios regulatory T cell (Treg) subsets**. **(A)** Representative flow cytometric plots showing PD-1 and CTLA-4 co-expression in different FoxP3 and Helios Treg subsets from healthy donor (HD)-peripheral blood mononuclear cell (PBMC), colorectal cancer (CRC)-PBMC, non-tumor-infiltrating lymphocytes (NILs), and tumor-infiltrating lymphocytes (TILs). Whisker plots comparing the relative levels of PD-1^+^CTLA-4^+^ cells **(B)** and PD-1^+^CTLA-4^−^ cells **(C)** in FoxP3^−^Helios^+^, FoxP3^+^Helios^+^, and FoxP3^+^Helios^−^ Treg subsets. **(D)** Absolute percentages of PD-1^+^CTLA-4^+^ and PD-1^+^CTLA-4^−^ in different FoxP3 (F) and Helios (H) Treg subsets in TILs.

**Figure 5:**
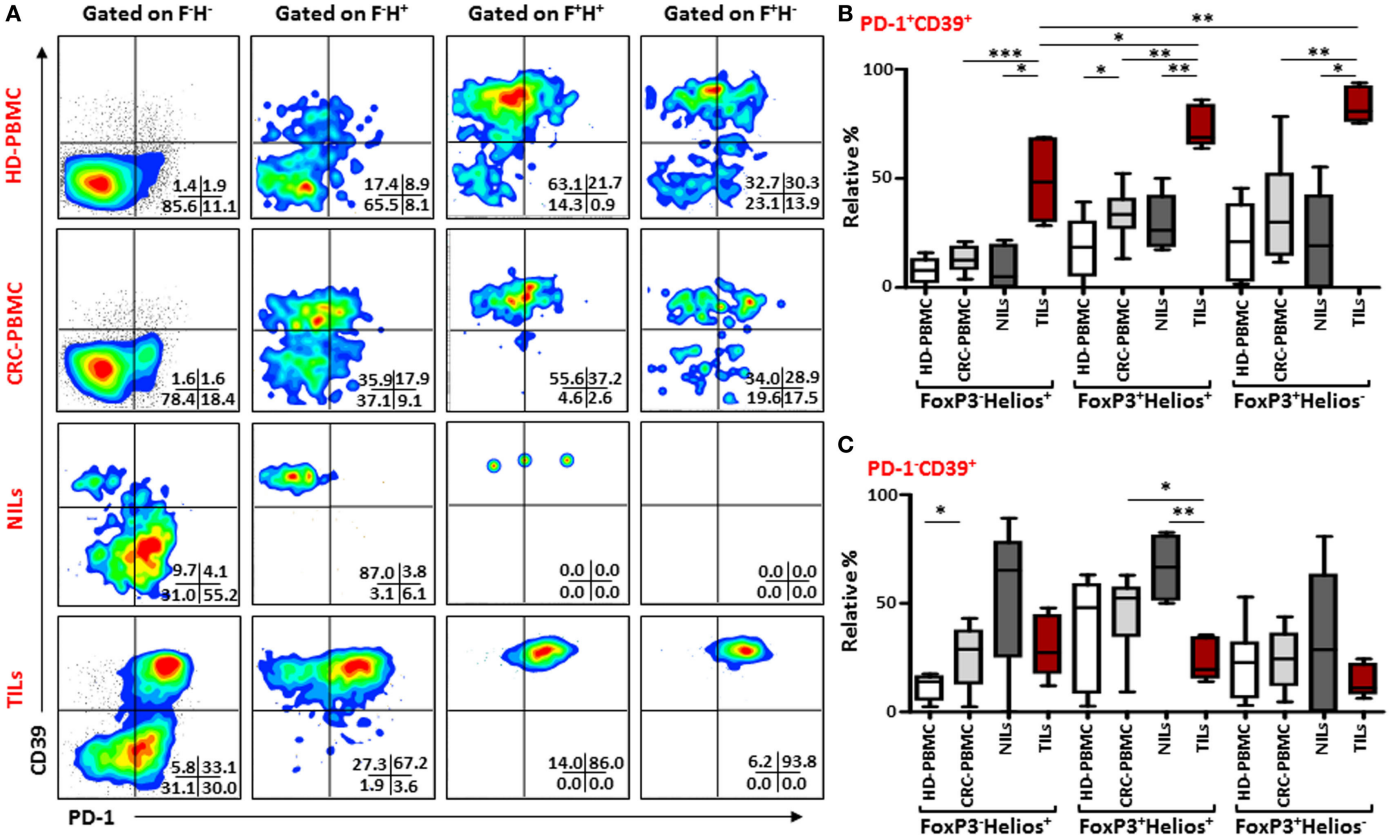
**PD-1 and CD39 co-expression in different FoxP3 and Helios regulatory T cell (Treg) subsets**. Representative flow cytometric plots showing PD-1 and CD39 expression in different FoxP3 and Helios Treg subsets from healthy donor (HD)-peripheral blood mononuclear cell (PBMC), colorectal cancer (CRC)-PBMC, non-tumor-infiltrating lymphocytes (NILs), and tumor-infiltrating lymphocytes (TILs) are shown in panel **(A)**. Whisker plots comparing the levels of PD-1^+^CD39^+^ cells **(B)** and PD-1^−^CD39^+^ cells **(C)** in FoxP3^−^Helios^+^, FoxP3^+^Helios^+^, and FoxP3^+^Helios^−^ Treg subsets.

Next, to further define these Tregs accumulated in TILs and the different markers they express compared to those in NILs and in peripheral blood circulation of CRC patients, we examined the co-expression of PD-1/CTLA-4 and PD-1/CD39 in different FoxP3/Helios Treg subsets. Representative flow cytometric plots of PD-1/CTLA-4 co-expression and PD-1/CD39 co-expression within different FoxP3/Helios Treg subsets in different groups are shown in Figures 4A and 5A, respectively. In TILs, PD-1 and CTLA-4 co-expression was observed in FoxP3^−^Helios^+^, FoxP3^+^Helios^+^, and FoxP3^+^Helios^−^ subsets, which were significantly higher compared with their levels in NILs and circulating PBMCs (Figure 4B). There were no significant differences in PD-1 and CTLA-4 co-expression levels within different subsets of FoxP3 and Helios in circulating PBMCs between HDs and CRC patients. However, TILs exhibited significantly higher levels of cells expressing PD-1, but not CTLA-4, in all the three FoxP3/Helios subsets, compared with NILs and PBMCs (Figure 4C). Within TILs, the highest levels of ICRs co-expression, in terms of relative percentages, were observed in FoxP3^+^Helios^+^ and FoxP3^+^Helios^−^ Treg subsets; however, when investigated using absolute percentages, FoxP3^+^Helios^+^ Treg subset had the highest levels of PD-1 and CTLA-4 co-expression followed by FoxP3^−^Helios^+^ subset, and these were significantly higher than their co-expression in FoxP3^+^Helios^−^ Treg subset (Figure 4D). Moreover, FoxP3^+^Helios^+^ Treg subset showed higher absolute levels of PD-1^+^CTLA-4^−^ cells than FoxP3^−^Helios^+^ and FoxP3^+^Helios^−^ Treg subsets (Figure 4D).

Similar results were observed when we investigated co-expression of PD-1 and CD39 within different FoxP3/Helios Treg subsets (Figure 5A). In TILs, majority of cells within FoxP3^−^Helios^+^, FoxP3^+^Helios^+^, and FoxP3^+^Helios^−^ subsets co-expressed PD-1 and CD39, and their levels were much higher in TILs, compared with NILs and PBMCs. Within TILs, the highest levels of PD-1^+^CD39^+^ cells were observed in FoxP3^+^Helios^+^ and FoxP3^+^Helios^−^ subsets (Figure 5B). Moreover, the level of PD-1/CD39 co-expressing CD4^+^FoxP3^+^Helios^+^ Tregs was significantly higher in peripheral blood of patients compared with HDs. Notably, in NILs, levels of cells expressing only CD39 within different FoxP3/Helios subsets were higher compared to TILs, but the difference reached significance only in FoxP3^+^Helios^+^ subset (Figure 5C).

## Discussion

In this study, we focused on phenotypic characterization of intratumoral Tregs in CRC patients and observed the presence of different immunosuppressive molecules including CTLA-4, PD-1, and CD39. CRC being an inflammatory disease is characterized by the accumulation of different types of immune cells within the TME. We observed significant accumulation of CD45^+^CD3^+^ cells within colorectal TT, which is in line with previous studies (35). Majority of these infiltrating CD3^+^ cells were CD4^+^, and this selective infiltration of CD4^+^ T cells within TT might be partly due to the requirement of CD4^+^ T cells for efficient priming and activation of CD8^+^ T cells (36). This also suggests a pro-tumorigenic function of infiltrating CD4^+^ T cells in CRC patients, as accumulation of CD8^+^ T cells would have indicated a tumor-specific immune response as a protective function against cancer. Moreover, the increase in CD45^+^CD3^−^ cells within TT indicated the presence of cells of myeloid origin and might be of importance in colorectal tumorigenesis. We have recently shown that granulocytic cells of myeloid origin were elevated in peripheral blood and the TME of this CRC patients’ cohort and their levels correlated with tumor stage and histological grades of the disease (32). These tumor-infiltrating CD4^+^ T cells expressed high CD25, which indicated the presence of Tregs, and this can be a result of conversion of CD4^+^CD25^−^ cells into CD4^+^CD25^+^ cells in which TGF-β is involved (37). These cells also expressed relatively higher levels of CD39 and LAP compared with NILs, indicating their suppressive characteristics.

Immune checkpoint receptors such as PD-1 and CTLA-4 not only play important roles in immune homeostasis, but these inhibitory pathways are also utilized by tumor cells for immune response evasion. In the present study, we observed relatively higher frequency of CD8^+^ T cells expressing PD-1 in TILs, indicating their “exhaustive state,” which might have rendered them unresponsive in mounting any cell-mediated immune responses against cancer and blocking PD-1/PD-L1 inhibitory pathway restored their functions (38). Zhang et al. (39) reported high PD-1 expression on dysfunctional CD8^+^ T cells from PBMC and tissue of human non-small cell lung cancer, and blocking PD-1/PD-L1 pathway using PD-L1-specific antibody partially restored proliferation and cytokine production in these cells. Dunne et al. (23) also observed a significant increase in PD-1 expressing CD8^+^ T cells within colorectal TT compared with adjacent NT. They also reported a similar increase in CD4^+^PD-1^+^ T cells in TT relative to NT, which we did not observe. However, a significant difference in PD-1-expressing CD4^+^ T cells between NILs and TILs was observed if absolute percentages were compared. Overall, these contrasting results indicate that PD-1 might downmodulate the activating signals in CD8^+^ T cells in TT, but its role in CD4^+^ T cells requires further investigation.

Majority of the studies have reported the expression of either CTLA-4 or PD-1 on immune cells in cancer; however, limited studies described the possible associations between ICR pathways in cancer. Duraiswamy et al. (40) showed the co-expression of PD-1 and CTLA-4 was associated with antigen-specific T-cell dysfunction in xenograft mouse model and reported that dual blockade of these ICR pathways restored T-cell functions. Here, we showed a high frequency of CD4^+^ T cells co-expressed PD-1 and CTLA-4 in TILs, which might synergistically dampen T-cell activation and function in CRC patients. The over-expression of CD39 but not PD-1 on CD4^+^ T cells in NILs suggested that CD39 or PD-1 alone might not contribute to tumor-specific immune suppression, and that co-expression of CD39 with PD-1 in TILs indicated a T-cell dysfunction mechanism used by tumor cells to evade host immune response.

The marked increase in CD4^+^FoxP3^+^ Treg levels in TT compared to NT and peripheral blood of CRC patients in our study was in accordance with previously published reports from other groups, where an expansion in Treg levels in TILs in various cancers including CRC was demonstrated (23, 26, 33). Other studies, however, also reported an increase in circulating Treg levels in CRC patients compared to HDs (24, 29), which was not observed in our patient’s cohort. This disparity could again be due to the limited sample size. A role of FoxP3-expressing Tregs has remained ambiguous in colorectal tumor progression, as some studies showed a protective role of FoxP3-expressing Tregs in CRC (27), while other studies have shown it to be associated with an adverse outcome of the patients (28, 29). The reasons for these discrepancies, though not clear, might be due to the methods employed for identifying Tregs in tissue, or due to the presence of functionally distinct subpopulations of Tregs with different levels of immunosuppression as reported by Miyara et al. (30). Another reason might be due to the presence of different immunosuppressive molecules such as Helios, PD-1, CTLA-4, and CD39 in Tregs. Helios is upregulated in Tregs (41) and is essential for the stable Treg inhibitory functions (10). Moreover, our group has recently suggested that FoxP3^+^Helios^+^ Tregs represent a functional subset with stronger suppressive characteristics (11). Consistent with these data, we observed a strong positive correlation between FoxP3 and Helios expression in TILs. Majority of FoxP3^+^ Tregs co-expressed Helios in these TILs, indicating these with greater immunosuppressive potential than either FoxP3 or Helios expressing Treg subset.

One of the reasons for Treg accumulation at tumor site can be due to the production of chemokines by tumor cells and stroma in the TME, which mediates Treg influx within the TT. Furthermore, the conversion of FoxP3^−^ cells into FoxP3^+^ cells in presence of TGF-β or increased proliferation of Tregs can lead to their expansion (42). Our data suggested that the TME might enhance the conversion of FoxP3^−^Helios^+^ Treg subset into FoxP3^+^Helios^+^ Treg subset as FoxP3^−^Helios^+^ subset constituted more than half of different FoxP3 and Helios expressing Treg subset in NT and peripheral blood of cancer patients, but within the TME, FoxP3^−^Helios^+^ comprised of around 30% of Treg population and FoxP3^+^Helios^+^ Treg subset increased significantly and constituted more than 54% of the population among the three Treg subsets.

Apart from CTLA-4 and PD-1 immunoregulatory pathways, FoxP3^+^ Tregs can also suppress effector T cell functions through CD39/CD73 pathway, which involves adenosine hydrolysis from ATP (19). Nikolova et al. (43) showed that blocking of CD39 using neutralizing antibodies attenuated immunosuppressive functions of Tregs. In our current study, CD39 but not PD-1 expression was observed in FoxP3^−^Helios^+^ and FoxP3^+^Helios^+^ Treg subsets in normal tissue, whereas within TT, co-expression of PD-1 and CD39 was observed in all the three Treg subsets, indicating that CD39 expression alone is not sufficient for Treg-mediated immune suppression, but its co-expression with PD-1 is required for these Treg subsets for tumor-specific immune suppression. Furthermore, these FoxP3^+^Helios^+^ and FoxP3^+^Helios^−^ Tregs also co-expressed PD-1/CTLA-4, suggesting that synergistic associations between different immune inhibitory molecules might regulate immunosuppressive functions of these Treg subsets.

Studies have shown that blocking CTLA-4 pathway resulted in reduction in Treg-mediated immunosuppression and hence activation and proliferation of T cells, whereas PD-1 pathway blockade removes the “exhaustive” state of T cells and restores their antitumor activity. Blocking only one of the ICRs might not be very effective in CRC patients (44). In a recent Phase I clinical trial with anti-PD-1 blocking antibody alone, no objective responses were seen in CRC patients (45). Additionally, CTLA-4 monotherapy was also not very successful in CRC models, but its potent activity was seen in combination with other therapies (46). Preclinical studies and initial clinical trials have shown that the dual blockade of these ICRs might result in more effective antitumor immune responses (47–49). Overall, we report the co-expression of different immunosuppressive molecules in FoxP3^+^Helios^+^ and FoxP3^+^Helios^−^ Treg subsets, which might synergistically dampen T-cell activation and function and suppress tumor-specific immune responses in CRC patients. The effect of these different Treg subsets on the clinical outcome could not be validated in our patients’ cohort due to limited sample size. Our data suggest that dual blockade of these immunosuppressive molecules might have the potential to be used for more effective strategy for inducing antitumor immune responses in CRC patients.

## Ethics Statement

Ethical approval (protocol no. 13/23-CRD 244/13) was obtained from Al Ain Medical District Human Research Ethics Committee. All participating individuals signed written informed consent prior to collection of samples. All experiments were performed in accordance with relevant guidelines and regulations.

## Author Contributions

ASSK performed experimental work, data analysis, and wrote the manuscript. ST performed experimental work and data analysis. EE conceived the idea, designed the study, obtained fund, analyzed and interpreted data, and wrote and revised the manuscript. HE contributed to sample collection and acquisition of patients’ clinical data. BA obtained funds and revised the manuscript. All authors were involved in the final approval of the manuscript.

## Conflict of Interest Statement

The authors declare that the research was conducted in the absence of any commercial or financial relationships that could be construed as a potential conflict of interest.
