# Supplementary material for: Intratumoral FoxP3+Helios+ Regulatory T Cells Upregulating Immunosuppressive Molecules Are Expanded in Human Colorectal Cancer
Source: Front Immunol. 2017 May 26;8:619. doi: 10.3389/fimmu.2017.00619 (PMC5445103; doi:10.3389/fimmu.2017.00619)
Supplement: Supplementary file 1 [file image_1.pdf]

Supplementary Figures

Supplementary Figure 1

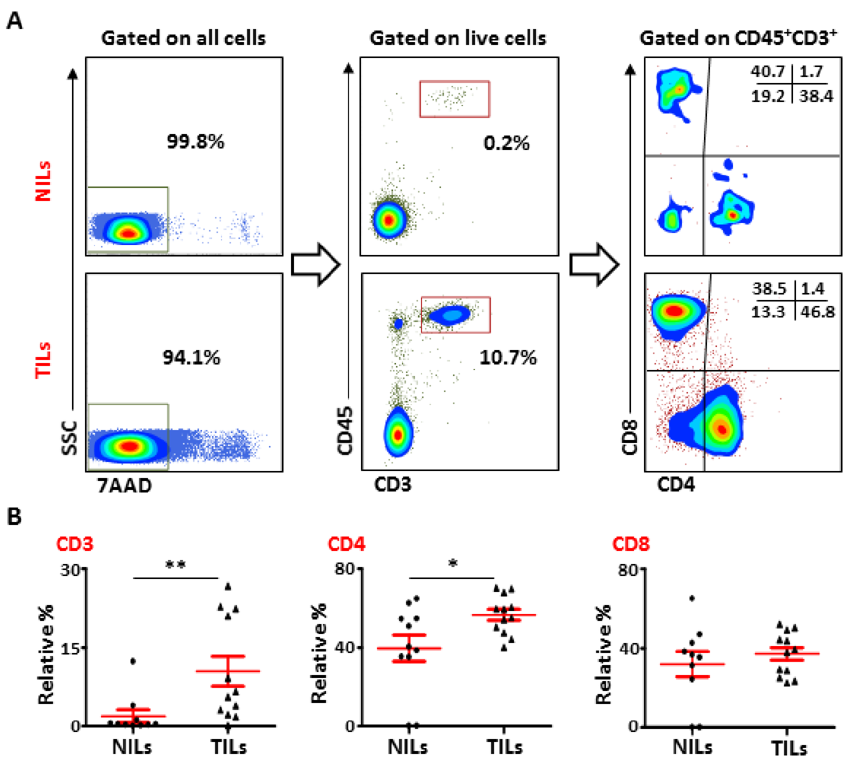

Supplementary Figure S1: T cell infiltration in tumor and non-tumor tissue in CRC. Freshly isolated immune cells infiltrating non-tumor (NILs) and tumor tissues (TILs) from 10 CRC patients were stained with 7AAD, CD45, CD3, CD4 and CD8 antibodies for identification of T cells and their subsets. Representative flow cytometry plots of surface staining from a cancer patient are shown in **A**. Live cells are gated first using 7AAD, followed by lymphocyte identification by CD45 and CD3 stainings. Different subsets of T cells were then characterized using CD4 and CD8 antibodies. **B.** Scatter plots showing the differences in tissue-infiltrating CD3, CD4 and CD8 between NILs and TILs.

Supplementary Figure 2

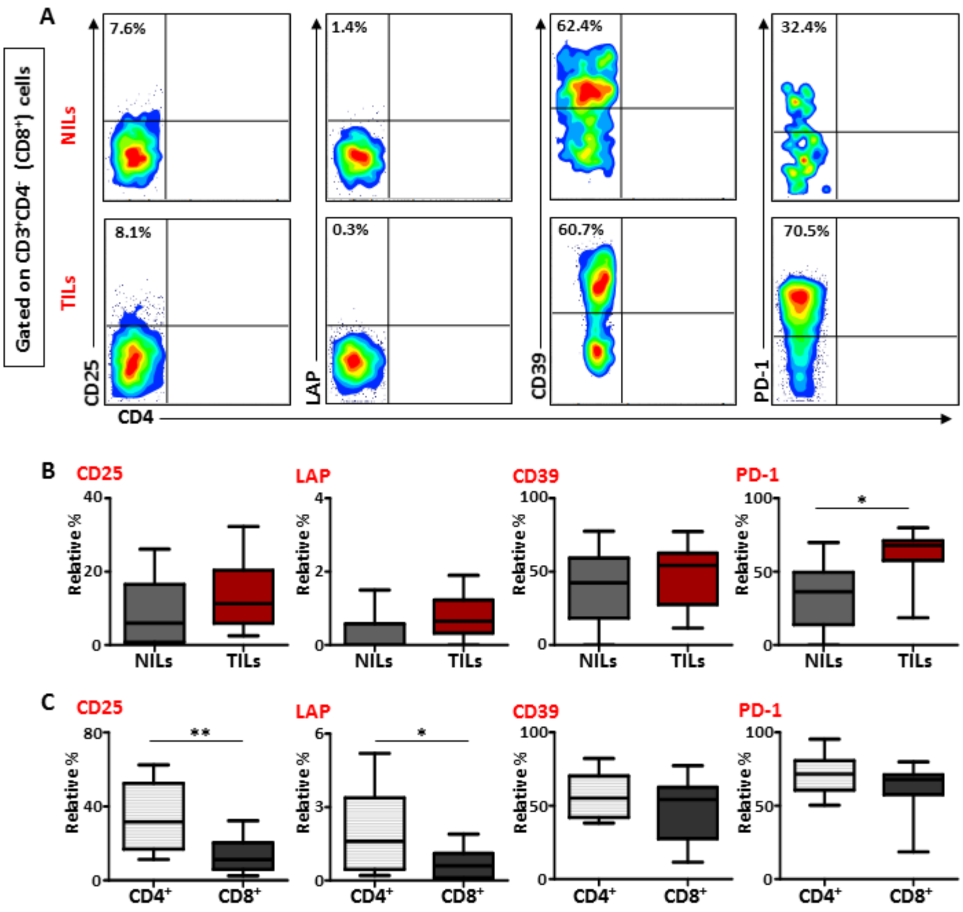

Supplementary Figure S2: Phenotypic characterization of CD8^{+} T cells in NILs and TILs. Freshly isolated NILs and TILs were stained for CD25, LAP, CD39 and PD-1 surface markers. Live cells were gated using 7AAD dye. Levels of these markers were calculated in CD3^{+}CD4^{-} (CD8^{+}) T cells. **A.** Representative Flow cytometric plots for CD25, LAP, CD39 and PD-1stainings in NILs and TILs from a cancer patient. **B.** Scatter plots comparing the differences between these surface markers in CD8^{+} between NILs and TILs. **C**. Scatter plots showing the differences in CD25, LAP, CD39 and PD-1 expression between CD4^{+} and CD8^{+} populations in TILs.

Supplementary Figure 3

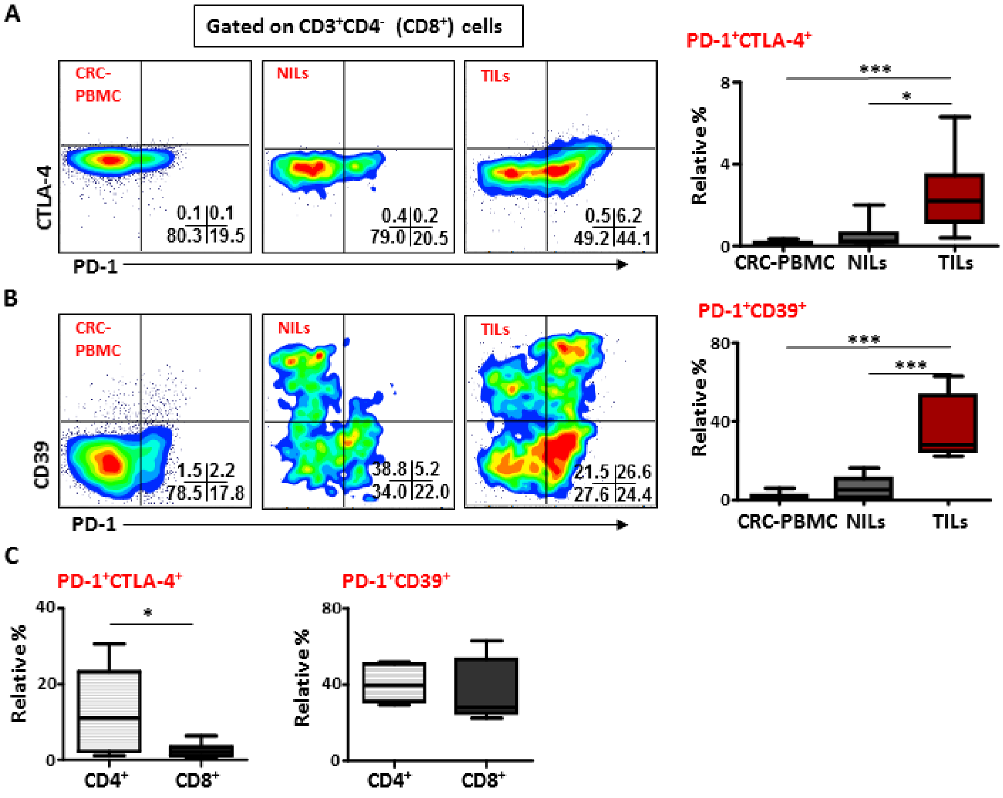

Supplementary Figure S3: Co-expression of PD-1/CTLA-4 and PD-1/CD39 in CD3^{+}CD4^{-} (CD8^{+}) T cells in patients PBMCs, NILs and TILs. CRC PBMCs, NILs and TILs were stained for CD3, CD4, PD-1 and CD39 followed by intracellular staining for CTLA-4. Live cells were gated using Fixable Viability Dye 660. Representative Flow cytometric plots showing different populations of PD-1 and CTLA-4 (**A)** and PD-1 and CD39 (**B**) in CD8^{+} T cells from CRC-PBMC, NILs and TILs are shown. **C.** Comparisons of PD-1/CTLA-4 and PD-1/CD39 co-expressions between CD4^{+} and CD8^{+} populations in TILs.
